# Embedding mRNA Stability in Correlation Analysis of Time-Series Gene Expression Data

**DOI:** 10.1371/journal.pcbi.1000141

**Published:** 2008-08-01

**Authors:** Lorenzo Farina, Alberto De Santis, Samanta Salvucci, Giorgio Morelli, Ida Ruberti

**Affiliations:** 1 Dipartimento di Informatica e Sistemistica “Antonio Ruberti”, Sapienza Università di Roma, Rome, Italy; 2 Istituto di Biologia e Patologia Molecolari, Consiglio Nazionale delle Ricerche, Rome, Italy; 3 Istituto Nazionale di Ricerca per gli Alimenti e la Nutrizione, Rome, Italy; University of California Santa Cruz, United States of America

## Abstract

Current methods for the identification of putatively co-regulated genes directly from gene expression time profiles are based on the similarity of the time profile. Such association metrics, despite their central role in gene network inference and machine learning, have largely ignored the impact of dynamics or variation in mRNA stability. Here we introduce a simple, but powerful, new similarity metric called *lead-lag R^2^* that successfully accounts for the properties of gene dynamics, including varying mRNA degradation and delays. Using yeast cell-cycle time-series gene expression data, we demonstrate that the predictive power of lead-lag R^2^ for the identification of co-regulated genes is significantly higher than that of standard similarity measures, thus allowing the selection of a large number of entirely new putatively co-regulated genes. Furthermore, the lead-lag metric can also be used to uncover the relationship between gene expression time-series and the dynamics of formation of multiple protein complexes. Remarkably, we found a high lead-lag R^2^ value among genes coding for a transient complex.

## Author Summary

Microarrays provide snapshots of the transcriptional state of the cell at some point in time. Multiple snapshots can be taken sequentially in time, thus providing insight into the dynamics of change. Since genome-wide expression data report on the abundance of mRNA, not on the underlying activity of genes, we developed a novel method to relate the expression pattern of genes, detected in a time-series experiment, using a similarity measure that incorporates mRNA decay and called *lead-lag R^2^*. We used the lead-lag R^2^ similarity measure to predict the presence of common transcription factors between gene pairs using an integrated dataset consisting of 13 yeast cell-cycles. The method was benchmarked against six well-established similarity measures and obtained the best true positive rate result, around 95%. We believe that the lead-lag analysis can be successfully used also to predict the presence of a common mechanism able to modulate the degradation rate of specific transcripts. Finally, we envisage the possibility to extend our analysis to different experimental conditions and organisms, thus providing a simple off-the-shelf computational tool to support the understanding of the transcriptional and post-transcriptional regulation layer and its role in many diseases, such as cancer.

## Introduction

Gene expression is a highly regulated process composed of two fundamental biological events: synthesis and degradation. Transcription regulation is achieved by modulating the frequency of transcription initiation and, although the most studied, this event represents just the first of the many complex stages leading to a mature mRNA. Recent experimental work is beginning to shed light on the complex architecture underlying mRNA degradation pathways by identifying the factors and enzymes involved. Therefore, it is now widely accepted that mRNA decay contribution to the control of gene expression is not simply a biological waste-disposal system, but a key player for the temporal coordination of cellular functions. Moreover, a number of highly complex and sophisticated specific mechanisms have been identified [1]. Such mechanisms include the interaction with mRNA binding proteins [2] and the nonsense-mediated mRNA decay pathway [3], both able to affect the accumulation of hundreds of transcripts.

Recent technologies, such as microarrays, are able to provide measurements of mRNA abundance over time under different experimental conditions. In order to decipher the intricate regulatory network underlying the highly coordinate cell behavior, effective computational methods have been developed to take advantage of gene expression data. The basic idea underlying such methods stems from the experimental observation that genes are organized in groups showing similar time profiles [4] (called “clusters”). These groups often share some common biological features, such as the same cellular function or the presence of a common motif at their promoter regions [5] where transcription factors (TFs) can bind and possibly turn them on or off in a coordinated manner, when needed. For this reason, it is now widely accepted that *co-expression* is a good indication for *co-regulation* [6]–[8], meaning that whenever two genes display similar time profiles it is likely that they are both targets of the same transcription factor(s). The search for co-regulated genes depends on association metrics used by clustering algorithms [5],[9],[10] and gene network inference algorithms [11]–[13]. Therefore, measuring the degree of co-expression of genes is a fundamental step for data analysis, and in fact, many similarity measures have been proposed in the literature [14]. Among those available to quantitatively measure simultaneous expression, we will refer to the usual R^2^ value obtained from a linear regression model between two given gene expression time profiles denoted by *m_A_*(*t*) and *m_B_*(*t*). Their co-varying degree is therefore measured as the fraction of the total variance explained by the regression *m_A_*(*t*) = *c*_1_ *m_B_*(*t*)+*c*_2_. Such coefficient, indicated in this paper as the *simultaneous* R^2^ of the corresponding gene pair, is the square of the Pearson correlation and takes values between 0 and 1.

In order to infer the gene regulatory network, several laboratories have combined microarray data with protein-DNA interaction data, taking advantage of ChIP-on-chip experiments [15]. Such studies have shown that the same transcription factor (or combinations of) may target genes with very different expression time profiles, even in the same experimental condition. For example, the targets of the yeast cell cycle transcriptional regulators MBF/SBF display expression peak times that span from early G1 to late S. Moreover, delays have been recently observed between putatively co-regulated genes [16],[17]. One fundamental biological mechanism underlying such temporal spread is certainly combinatorial regulation of transcription factors. In fact, various TFs can modulate target response by cooperating or competing for DNA binding. Consequently, new computational techniques have recently appeared in the literature to tackle this problem [18]–[25]. However, combinatorial regulation is not the only mechanism responsible for peak time delay, as other regulation layers are active throughout transcript life and impact its abundance over time. One such additional regulation layers is certainly the post-transcriptional one, that is the stability properties of transcripts that may specifically contribute to the determination of their timing and amount during cell response to various internal and/or external signals. Strikingly, recent genome-wide measurement of the yeast transcripts half-lives [26],[27] has shown functional specificity in mRNA decay. Together, these results pointed to a general relationship between physiological function and mRNA decay rate thus providing strong evidence that precise control of mRNA turnover is a fundamental feature of gene expression programs in yeast [26] and in many other organisms.

Here we focus on the development of a novel computational tool aiming to uncover co-regulated genes through transcriptional and post-transcriptional regulatory mechanisms. To this purpose, starting from the computational approach developed by Farina *et al.* [28], we introduce a new relationship between gene pairs, called *lead-lag relationship*. The term “lead-lag” has been taken from the field of control systems engineering where the same relationship holds between the input and the output of the so called “lead-lag compensator”, which is the fundamental building block for the design of automatic control systems [29]. In a biological perspective, the lead-lag relationship should be referred to genes under a common regulatory signal (“input”) involved in the same biological function (“output”) as, for example, in the dynamic multi sub-units complex formation [30],[31]. Using yeast cell-cycle time-series gene expression data, we demonstrate that this new similarity metric is able to capture the dynamics of gene expression, including varying mRNA stability and delays. Thus, the predictive power of lead-lag R^2^ for the identification of co-regulated genes is significantly higher than that of standard similarity measures, allowing the selection of a large number of entirely new putatively co-regulated genes. Furthermore, the lead-lag metric can also be used to uncover the relationship between gene espression time-series and the formation of protein complexes.

## Results/Discussion

### Specific Features of Transcript Degradation Regulation Versus Transcription Regulation

**Figure 1:**
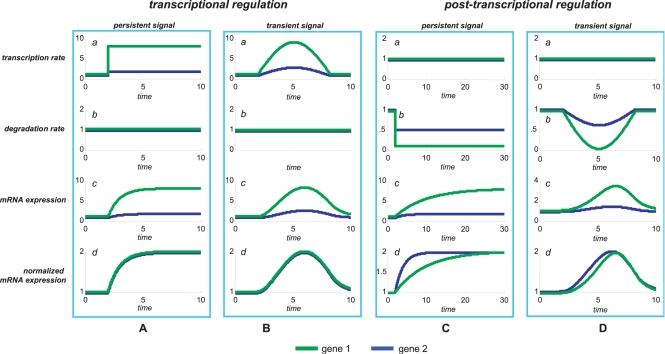
Combination of transcriptional and post-transcriptional regulation. Gene expression time profiles obtained by simulations using various types of regulation of the transcription rate and degradation rate. Panels A and B refer to the situation in which two genes have the same constant degradation rate but different transcription rate signals (persistent or transient). Panels C and D refer to the situation in which two genes have the same constant transcription rate but different degradation rate signals (persistent or transient). Normalized time profiles are linearly scaled such that their values remain bounded between 1 and 2, *i.e.* by setting to 1 the lowest value and to 2 the highest value so that the peak-to-peak amplitude is set to 1.

To clarify the specific features of gene regulation at the mRNA stability level, it is worth thinking of the case when two genes are turned on at the same time by the same transcriptional signal, and the newly synthesized transcripts of both genes are degraded at the same rate. Consequently, differences in their gene expression profile will be determined only by the response of the two genes to the transcriptional signal (*i.e.* different affinities of the transcription factor to promoter regions). A computer simulation of this situation is depicted in Figure 1A where two genes are expressed following a first-order kinetics (see Text S1. for details on the equations used for the simulation). The transcription is turned on at the same time for both genes but with a different rate: the first gene is transcribed more rapidly than the second one (Figure 1Aa). Their degradation rate is the same (Figure 1Ab) and therefore the two gene expression profiles differ only for the magnitude of the response, whereas preserving the shape of the curve (Figure 1Ac). In this case, the normalized time profiles are identical (Figure 1Ad) and therefore the simultaneous R^2^ is maximal (R^2^ = 1). Indeed, the “converse” situation is very different. Figure 1Ca–b illustrates the case in which the two genes are transcribed at the same rate while their degradation decreases at the same time but with a different rate. The two profiles do not have the same shape (Figure 1Cc). As a consequence, the corresponding simultaneous R^2^ will not be maximal (R^2^<1) as can be seen from differences in the normalized profiles (Figure 1Cd).

Such considerations illustrate that the impact of stability regulation on time profiles is quantitatively and – most importantly – *qualitatively* different from that of transcription regulation. It is therefore not surprising that specific systems biology computational tools have begun to appear in the literature [28],[32]. The different impact of mRNA stability regulation versus transcription regulation results from the fact that the rate of mRNA degradation is proportional to the substrate concentration but the rate of production is *not* [33]. Such behaviour is reasonably well captured by a first order rate equation. In fact, messengers half-lives are experimentally measured usually by fitting a single exponential decay function to the time profiles observed after transcriptional shut-off [26].

Another important issue is that the differences of transcription rate regulation with respect to degradation rate regulation cannot be clearly seen by simply looking at the long term behavior of the response, *i.e.* at steady state values. In fact, the final amount of mRNA upon a prolonged regulatory signal equals the ratio transcription rate/degradation rate so that, from this perspective, a *N*-fold increase of transcription rate is equivalent to a *N*-fold decrease in degradation rate (and viceversa). An example of such behavior can be seen by comparing Figure 1Ac with Figure 1Cc: the steady state values are the same in both cases but the overall shape of the response (its “dynamics”) is very different.

**Figure 2:**
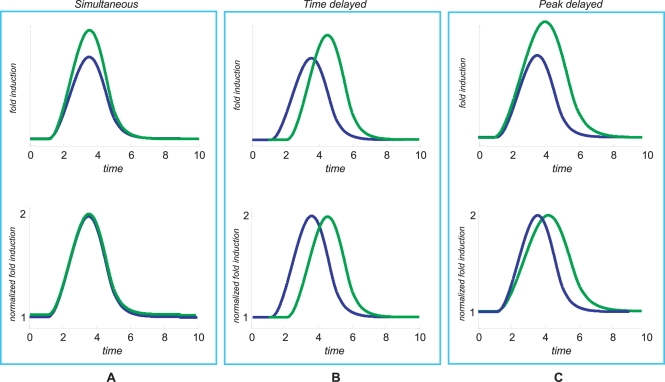
Relationships between gene expression time profiles. Typical behaviors of genes related by a simultaneous (Panel A), time-delayed (Panel B) and peak-delayed (Panel C) expression pattern. The time delayed profiles may be the result of different switching times in transcriptional activation whereas the peak delayed profile may be the result of the same transcriptional signal and different mRNA stabilities. Normalization is performed by setting to 1 the lowest value and to 2 the highest value so that the peak-to-peak amplitude is set to 1.

Such “loss of correlation” phenomenon due to differential stability regulation can be further understood by considering a time varying rates, resulting in a transient mRNA time profile, as shown in Figure 1Ba–d and 1Da–d. Again, an increase in the rate of transcription results in an increased response displaying a highly correlated temporal profile (Figure 1Bd), whereas a decrease in the degradation rate results in a low correlated temporal profile with a shift in peak time, as shown by Figure 1Da–d. It is plain that, by combining time varying transcription and degradation rates, a large variety of dynamic time patterns may be generated. It is important to note that peak timing regulation may also stem from time delays, as shown in Figure 2B, which can be generated by different biological mechanisms such as transcriptional combinatorial regulation, cascade regulations [34], feedforward motifs and single input motifs [35]. Time delays in gene expression data have been studied using delay correlation analysis [16],[36],[37].

The scenario depicted above naturally leads to the possibility that co-regulation may involve both the transcriptional and post-transcriptional machinery. Therefore, a large variety of temporal profiles can be obtained by combining any of those shown in Figure 2.

### The Lead-Lag Relationship

In this paper we consider a novel relationship between gene expression time profiles which includes also the possible presence of mRNA stability variations as a further mechanism to modulate transcript abundance over time. Such new coordinated relationship will be called *lead-lag relationship*. Such terminology is borrowed from the field of system and control engineering where it refers to the basic building block for the realization of a regulatory device able to provide optimal properties to a given process and called “lead-lag compensator” [29]. In order to identify lead-lag relationships, we propose a quantitative measure between gene expression time profiles, called *lead-lag R* ^2^, able to incorporate in a single parameter such relationship and consequently potentially enhancing the predictive power of gene expression analysis for the identification of putatively co-regulated genes. In fact, we aim to study here the possibility that an high lead-lag R^2^ between expression time profiles of two given genes is a good indication for the presence of a common regulation mechanism.

The lead-lag R^2^ is quantitatively defined by a linear multiple regression model among the two given gene expression time profiles *m_A_*(*t*) and *m_B_*(*t*) and the area under curve until time *t* (*i.e.* their time integral over time):
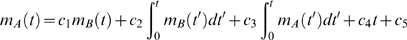
and measured by the *lead-lag* R^2^, that is the fraction of the total variance explained by the above multiple regression model. Such coefficient is computed directly from at least 6 time points of gene expression data and takes values between 0 and 1. The rationale behind such new relationship stems from a simple mathematical model conceived to capture, from gene expression time series data, those genes which are co-regulated at the transcriptional level having an equal or different mRNA stability.

It is worth noting that the simultaneous relationship is also a particular lead-lag relationship (just set *c_2_* = *c_3_* = *c_4_* = 0) so that the magnitude of the lead-lag R^2^ is always larger or equal than that of the simultaneous R^2^. In the following we will show that the magnitude of the increase from simultaneous R^2^ to lead-lag R^2^ is specific for each gene pair and that it is statistically correlated both to the presence of a common transcriptional signal and to differences between the half-lives. More details of the lead-lag R^2^ and its numerical computation are given in the Materials and Methods section.

### Predicting Co-Regulation from Lead-Lag Relationships

**Figure 3:**
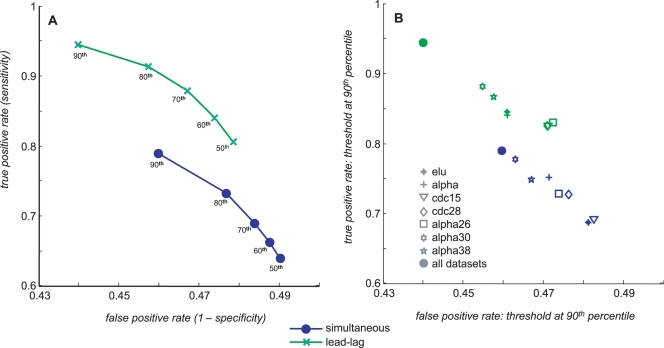
Predicting co-regulation: ROC curves. The ROC curves for co-regulation prediction were computed using 10 transcription factors involved in the cell cycle and assuming as true targets the DNA binding data provided by MacIsaac *et al.* [15], with *p*-value for binding 0.001. Two varying thresholds have been used for constructing the ROC curves; the higher percentile *p* (ranging from 50% to 90%) for the prediction of co-regulation, and the symmetric (100−*p*) percentile (ranging from 50% to 10%) for no co-regulation. Panel A shows ROC curves corresponding to R^2^ values averaged over all the 7 available datasets. Numbers below marks (circles and crosses) indicate the percentile of the distribution of R^2^ values used for selecting the lower and upper thresholds. Panel B shows ROC curve for each dataset obtained using as a threshold for co-regulation prediction only the 90^th^ percentile of the corresponding distribution.

The mathematical model used to define the lead-lag R^2^ is based on the assumption that co-regulated genes have the same transcriptional signal (promoter activity) and equal or different transcript stabilities. Consequently, we postulated that two given genes showing a lead-lag relationship (namely, with high lead-lag R^2^ values) are likely to be regulated by common transcription factors. To test this hypothesis, we selected a list of 1159 genes indicated as cell-cycle regulated in at least one out of six yeast genome-wide studies [38]. We then used a large integrated dataset of yeast cell-cycle data generated by three independent groups using different synchronization methods and composed of 7 datasets (13 cell cycles for each gene, see Materials and Methods for details). We considered as “gold standard” the transcriptional regulatory network recently published by MacIsaac and collegues [15]. Such reconstructed network is very reliable since the authors combined complementary strategies to improve the ability to identify the specificity of transcriptional regulators from genome-wide chromatin immunoprecipitation data. The Mc Isaac *et al.* dataset consists of a list of targets for 203 TFs using different conservative criteria. Among those available 203 TFs, we selected a *p*-value for binding of 0.001 obtaining a list of 3107 genes, containing 660 of the genes in the list of the cell cycle regulated ones. We then choose the 10 TFs widely recognized as having a fundamental role during the cell cycle [39]: SWI4, SWI6, MBP1, NDD1, FKH1, FKH2, MCM1, ACE2, SWI5 and YOX1. Using this data, we could assess the effectiveness of our approach by computing true and false positive rates and ROC curves. To this end, we evaluated the lead-lag R^2^ for each gene pair in the dataset (*N*(*N*−1)/2 pairs, *N* = 660) and considered as putatively co-regulated those pairs whose R^2^ values were over a threshold *t_high_* and, as putatively non co-regulated, those pairs whose R^2^ values were below a threshold *t_low_*. Gene pairs with scores between thresholds were not considered. In order to construct a ROC curve we used varying thresholds: as an upper threshold *t_high_* for co-regulation we selected the value corresponding to percentiles *p* ranging from 50^th^ to 90^th^ with a step of 10 and, as a lower threshold *t_low_* for non-coregulation, we selected the value corresponding to the “symmetric” percentile 100−*p*. For each threshold we could compute true positives, true negatives, false positives, false negatives and therefore construct a ROC curve (Figure 3A, green plots) where all the R^2^ values have been averaged over the 7 datasets. The average dataset has been constructed by computing the R^2^ values for each cycle and for each dataset, for a total amount of 13 cycles. The mean R^2^ value for each genes pair was obtained by computing the mean of the 13 available values. In case of missing data in the original dataset, computation of the mean R^2^ value was performed only when at least 8 out of 13 cycles were available. Each class of putatively co-regulated gene pairs was obtained by selecting those pairs exceeding the upper thresholds corresponding to the percentiles from 50^th^ to 90^th^ with a step of 10 of the R^2^ distribution. Therefore, true positives are those pairs of the class having at least one common transcription factor according to the Mc Isaac *et al.* dataset (*p*-value for binding<0.001), whereas false positives are those pairs in the class without a common transcription factor (*p*-value for binding>0.001). Analogously, true negatives and false negatives were computed within the class of gene pairs having the lower thresholds corresponding to the percentiles from 50^th^ to 10^th^ with a step of −10 of the R^2^ distribution.

To evaluate the performance of predictions obtained with the lead-lag R^2^ we repeated the same analysis using the simultaneous R^2^ as a similarity measure between two given genes (Figure 3A blue plots). The results clearly show that the lead-lag R^2^ certainly outperforms standard analysis based only on simultaneous relationships, increasing the true positive rate from 80% to 95%. The fraction of false positives also slightly decrease but remains relatively high possibly due to the fact that we have considered highly conservative criteria and selected the targets of only those 10 transcription factors having a major regulatory role during the cell cycle. It is worth noting that the performances on the average dataset are much better than the average of all performances (see Figure 3B) thus showing that an integrative approach using multiple independent datasets is always the best choice, whenever applicable. Moreover, given the large number of datasets considered, we can also conclude that the results obtained are largely independent of the noise and the stress response induced by the synchronization methods. Finally, we note that the above results remain valid even if we consider the transcriptional network presented by McIsaac *et al.* [15] using different selection criteria for DNA binding (see Text S1).

### mRNA Half-Lives and Lead-Lag R^2^

The peculiarity of the lead-lag relationship between two given genes relies on the presence of a common regulatory signal driving the expression of transcripts with equal or different mRNA half-lives. For this reason, we investigated whether co-regulated gene pairs having an high lead-lag R^2^ values are significantly enriched with differential transcript's stabilities. Half-life values are not available during the cell cycle and in the same experimental conditions used for establishing cell synchronization. Nevertheless, genome-wide half-lives data for un-synchronized cells were published recently by Wang *et al.* [26]. Using DNA microarrays, the authors precisely measured the decay of each yeast mRNA in YPD medium, after thermal inactivation of a temperature-sensitive RNA polymerase II. Such half-life measurements were not obtained during the cell cycle, so that we do not expect an exact agreement with the actual ones. Nevertheless, by considering a large number of gene pairs (16740) it appears reasonable that, on average, the half-life ratios between gene pairs may not vary significantly. Therefore, we used such available data for a statistical evaluation of the presence of gene pairs with high lead-lag R^2^ values with respect to the simultaneous R^2^ among those co-regulated pairs having large half-life ratios.

To this end, we considered all possible gene pairs having, at least, one common transcription factor according to the MacIsaac *et al.* dataset [15] using a *p*-value for binding less that 0.001 and considered five half-life ratio bins: less than 2-fold, from 2-fold to 3-fold, from 3-fold to 4-fold, from 4-fold to 5-fold and more than 5-fold. We computed the simultaneous R^2^ and also the difference between the lead-lag R^2^ and simultaneous R^2^ for all the gene pairs in each of the half-life bins. Such difference is used in order to select that part of the lead-lag R^2^ value which is not due to the simultaneous espression of the gene pair. Therefore, we got a distribution of values for each half-life ratio bin and computed the corresponding mean value and standard deviation.

**Figure 4:**
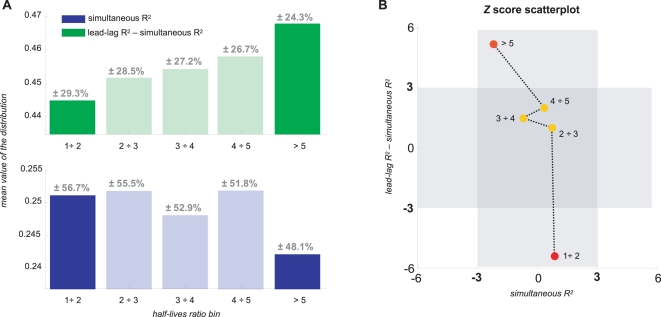
Sample gene pairs distribution of simultaneous R^2^ and lead-lag R^2^ minus simultaneous R^2^ versus half-life ratios. Panel A shows the bar plot of the mean values of the R^2^ distributions for co-regulated pairs. On the top of each bar is indicated the standard deviation as a percentage of the mean value. The difference between the means of the first and the last bin of the lead-lag R^2^ minus simultaneous R^2^ is significant according to a *t*-test with a confidence level of 95%, (*p*-value 10^−9^) and the difference between the means of the first and the last bin of the simultaneous R^2^ is mildly significant according to a *t*-test with a confidence level of 95%, (*p*-value = 0.03). According to the *t* test, no other difference is significant. The number of gene pairs contained in each bin are: 9128, 3726, 1707, 919 and 1260, respectively. Blue bars indicate the distribution of simultaneous R^2^ whereas green bars indicate the distribution of lead-lag R^2^ minus simultaneous R^2^. Panel B shows the scatterplot of the *Z*-scores corresponding to 100000 randomizations of the half-life measurements. Whereas the *Z* score of the simultaneous R^2^ does not show any significant change after half-life ratios randomization for each bin, the *Z* score of the lead-lag R^2^ minus the simultaneous R^2^, does show a significant change in the first and the last bin.

Figure 4 shows the results of the above described computation. Figure 4A makes clear that the highest and the lowest half-life bin display very different lead-lag minus simultaneous R^2^ mean values. To further support this feature, we performed a *t* test and found that the increase of the mean value of the distribution in the first and the last bin of the lead-lag R^2^ minus the simultaneous R^2^ is indeed significant (95% confidence level, *p*-value 10^−9^). The simultaneous R^2^ also shows a mildly significant decrease (95% confidence level, *p*-value 0.03) of the mean values between the first and the last bin. To further evaluate the statistical significance of this analysis we computed the *Z*-score corresponding to 100000 randomizations of the half-life measurements. The results are shown in the scatterplot of Figure 4B and they provide computational evidence that the lead-lag R^2^ of gene pairs is statistically correlated to their half-life ratios. In fact, a high positive *Z*-score (about 5) corresponds to the highest half-life ratio bin and a negative *Z*-score (about −5) corresponds to the first half-life ratio bin. On the other hand, *Z* scores for the simultaneous R^2^ are all within the values −3 and 3 and therefore the observed difference of the mean values between the first and the last bin is not significantly affected by the randomizations. This scenario is consistent with the biological process underlying the mathematical model used to define the lead-lag R^2^ thus showing that our analysis well captures the effects of post-transcriptional control on gene expression time profiles during the cell-cycle.

### Comparison to Other Similarity Measures

**Figure 5:**
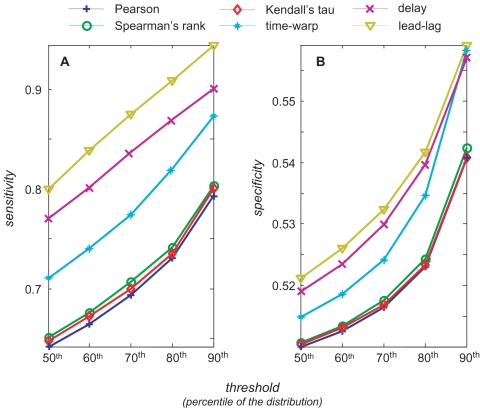
Performance comparisons of similarity measures for co-regulation prediction. The similarity measures considered for comparisons are: the square of Pearson, Spearman's rank, Kendall's tau, time-warped, time-delayed and lead-lag correlation. Sensitivity (Panel A) and specificity (Panel B) are shown as a function of the chosen percentile threshold. The results obtained using the for cosine correlation are not reported since it performed as a random choice.

The results presented so far have clearly shown that lead-lag correlation analysis outperforms the usual simultaneous correlation analysis (squared Pearson coefficient) for the prediction of co-regulation, *i.e.* the presence of a common transcription factor, from gene expression time profiles. As previously discussed, truly co-regulated genes do often display large differences of gene expression time profiles, *e.g.* peak shifts, delays or other kinds of nonlinear relationships. In this paragraph, we consider other similarity measures relevant to the analysis of gene expression data and compare their performances with those obtained using the lead-lag R^2^. In particular, we used 5 similarity measures other than the lead-lag: Spearman's rank, Kendall's tau, cosine, dynamic time-warped and time-delayed correlation, all squared to capture inverted relationships also. Spearman's rank, Kendall's tau and cosine correlation are the most common choices for the analysis of gene expression data in the presence of nonlinear relationships between time series, but they do not take into account the time ordering of data. By contrast, time-warped and time-delayed correlation have been specifically developed to analyze gene expression time profiles. The time-delayed correlation analysis has been proposed by Schmitt *et al.* [37] where, for any genes pair, a R^2^ value is obtained by selecting the highest simultaneous R^2^ over all admissible time delays between profiles. The dynamic time-warped correlation has been recently used by Aach and Church [40] and Hermans and Tsiporkova [41] for the alignment of gene expression time series obtained in experiments using different cell synchronization methods. These two works are both based, for gene-to-gene comparisons, on the Dynamic Time Warping (DTW) algorithm developed by Sankoff and Kruskal [42]. Accordingly, we defined a time-warped R^2^ by selecting the highest simultaneous R^2^ over all the possible time warped paths. For any similarity measure, we performed the same analysis reported in a previous section using the same data, and the results are shown in Figure 5 where sensitivity (panel A) and specificity (panel B) are reported for each threshold.

First of all, the cosine correlation analysis produces the poorest performances, very close to a random choice, and therefore such similarity measure is not reported in Figure 5. On both panels we note that simultaneous, Spearman's rank and Kendall's tau provide comparable results which are clearly below the performances of the group of methods which take into account the time ordering of data. In this group the lead-lag correlation analysis shows the best performances, both in terms of sensitivity and specificity.

### Examples of Lead-Lag Analysis Using Yeast Cell Cycle Gene Expression Data

In this section we present some examples of “typical” lead-lag relationships using the most recent yeast cell cycle data [43] and discuss their biological relevance. The complete list of gene pairs exceeding the 95^th^ percentile of the distribution for each of the R^2^ values considered in this paper is provided in the supporting information file Text S1.

#### Key cell cycle regulators under common transcription factors

**Figure 6:**
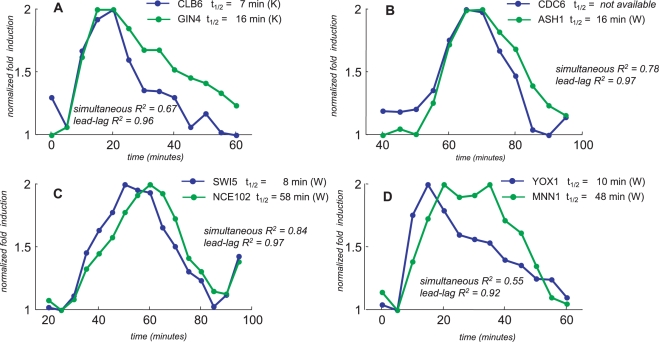
Examples of lead-lag relationships of key cell cycle regulators. The gene pairs reported in the figure are: CLB6/GIN4 (Panel A), CDC6/ASH1 (Panel B), SWI5/NCE102 (Panel C) and YOX1/MNN1 (Panel D). Gene expression time profiles are taken from Pramila *et al.* [43], alpha_38 dataset and the expression values are normalized with respect to peak-to-peak amplitude. Each half-life dataset is indicated in brackets: “W” denotes the Wang dataset [26], “K” denotes the Kuai dataset [45].

The budding yeast cell cycle is characterized by consecutive waves of expression of key regulators such as cyclins and transcription factors [44]. CLB6, a G1/S-phase cyclin, has a lead-lag relationship with GIN4 as shown in Figure 6A, a gene encoding a key component involved in transitioning to the next stage of the cycle [34]. The lead-lag relationship suggests the presence of a common transcription factor and, consistently, the two genes are both targets of the transcription factor complex MBF/SBF according to ChIP experiments [15]. Moreover, the time profiles shown in Figure 6A indicate also the possibility that the transcriptional signal is turned on and then quickly turned off, so that the subsequent behaviour of the two genes is mainly determined by the degradation process alone. Accordingly, transcripts stabilities – as measured after transcriptional shut-off [45] – significantly differ in value.

Cell Division Cycle 6 (CDC6) is a component of the pre-replicative complex essential for the initiation of DNA replication, normally expressed at the end of mitosis. It has a lead-lag relationships with ASH1 (Figure 6B) which encodes a GATA-like transcription factor localized at daughter cells where it serves to repress the late G1-specific transcription of HO and preventing mating-type switching [46]. Our analysis suggests the presence of a common regulatory signal. In fact, both genes are key regulators of separate biological processes that are simultaneously activated by the SWI5 transcription factor [15],[47],[48]. Moreover, our analysis also suggests that the CDC6 transcript is fairly unstable. Consistently, the CDC6 protein is unstable [47].

SWI5 encodes a key transcription factor that activates transcription of genes expressed at the M/G1 boundary and in G1 phase of the cell cycle. NCE102 is a non-classical export protein involved in alternative clearance/detoxification pathway to eliminate damaged material [49]. They display a lead-lag relationship (Figure 6C) and, in fact, they are both targets of the M-phase activating complex FKH2/NDD1 according to ChIP experiments [15] and large differences of their half-life values are observed after transcription inhibition [26]. The gene expression profiles shown in Figure 6C reflect the prototypical situation of peak delay depicted in Figure 2C.

YOX1 is a transcription factor involved in the repression of ECB acitivity [46] thus contributing to move the cycle forward. YOX1 shows a lead-lag relationship with MNN1 (Figure 6D), a gene encoding a cell wall glycoprotein [50]. Consistently, they are both targets of SBF according to ChIP experiments [15] and the transcripts have different half-life values [26]. Moreover, looking at the time profiles depicted in Figure 6D, one may argue that during the second half of the cycle another transcription factor is active at the MNN1 promoter.

All the above examples consist of pairs of genes that are under the control of the same transcription factor and that show differential mRNA stability values consistent with their lead-lag relationship (except for CDC6 transcript whose experimental half-life is not available). Moreover, it is worth noting that large differences in half-lives value (as in the cases shown in figure 6C and 6D) significantly affect the overall time profiles producing also an evident peak shift.

Finally, it is worth noting that the lead-lag relationship is symmetrical and, therefore, it does not provide information about which gene is “lead” and which is “lag”. However, such information can be easily obtained by visual inspection. In fact, from Figure 6A, the lead gene is the one with the steepest decaying profile having, consistently, a smaller half-life. Moreover, from Figure 6C, one can see that the gene with the larger half-life displays a delayed peak and, therefore, it corresponds to the lag gene.

#### Dynamic formation of the replication complex

Many studies have focused on the relationship between gene expression time courses and the formation of protein complexes. Interestingly, Jansen *et al.* [31] suggested to classify protein complexes as either *permanent* or *transient*, with permament ones being maintained through most cellular conditions. They also found that, generally, permanent complexes tend to have simultaneously correlated gene expression while transient ones do not. Moreover, they also noted that subunits of the same protein complex may show significant simultaneous expression. In particular, they studied gene expression of the replication complex in yeast and found a very low simultaneous correlation among subunits, not significantly different from a random control [31]. However, they also found two sub-complexes – the MCM complex and the DNA polymerases δ and ε complex – showing much greater simultaneous correlation.

**Figure 7:**
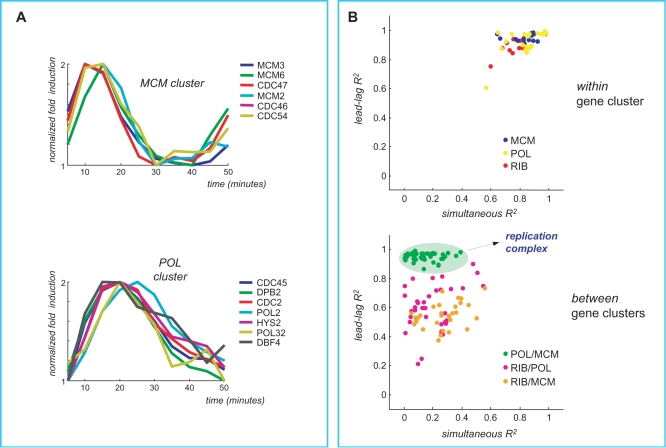
Lead-lag analysis of gene expression profiles of two components of the replication complex. The MCM cluster is composed by the time course of genes belonging to the MCM protein complex and the POL cluster by those belonging to the DNA polymerases δ and ε complexes. Panel A shows gene expression time profiles during one cell cycle ([43], alpha38 dataset) of the two groups of genes. In Panel B the scatterplots are computed for simultaneous and lead-lag R^2^ by considering within pairs for each cluster (MCM and POL) and between pairs (MCM/POL). The between pairs MCM/POL display a low value of simultaneous R^2^ and an high value of the lead-lag R^2^ consistently with the formation of the MCM/POL complex. Panel B also shows the results on a negative control composed by a group of simultaneously expressed genes encoding proteins that belong to the cytoplasmic ribosomal large subunit (RIB cluster). The between pairs MCM/RIB and POL/RIB display low values of both simultaneous and lead-lag R^2^ consistently with the absence of any interaction or co-regulation between the ribosomal proteins and the replication complex.

Using gene expression time profiles during one cell cycle ([43], dataset, alpha_38 time series) for the genes encoding MCM proteins (MCM cluster) and DNA polymerases and ε (POL cluster), we computed simultaneous and lead-lag R^2^ and the scatterplots of the resulting values for gene pairs belonging to the two different sub-complexes are shown in Figure 7, panel B. As a negative control we used a group of 5 simultaneously expressed genes (R^2^>0.7) coding for proteins of the cytoplasmic ribosomal large subunit (RPL4A, RPL4B, RPL1A, RPL1B, RPP0 denoted by RIB cluster). Ribosomal proteins are under the transcriptional control of IFH1/FHL1 [51],[52] whereas the replication complex is regulated by the transcription factors MBF/SBF [51]. The scatterplot reported in Figure 7 of the simultaneous *vs.* lead-lag R^2^ values shows that, whereas the POL/MCM pairs display high values of lead-lag R^2^ and low values of simultaneous R^2^, the control pairs POL/RIB and MCM/RIB display a very different pattern spread over a larger range thus denoting the absence of any meaningful relationship.

Figure 7 makes very clear that the gene expression of the MCM and the DNA polymerases δ and ε subcomplexes is significantly simultaneously correlated within the same group whereas such correlation dramatically drops if we consider pairs of genes belonging to different subcomplexes. In fact, the sample distribution of the simultaneous R^2^ between the two clusters is spread over the range [0,0.5] thus showing the absence of any significant level of simultaneous correlation. By contrast, the between clusters lead-lag R^2^ histogram is concentrated in the highest part of the range close to 1. The high values of the lead-lag R^2^ strongly suggest that mRNA stability may play a fundamental role in the dynamic formation of multiple protein complexes. Accordingly, the average half-life measured after transcriptional shut-off of the MCM group is 14±6 min (the “lead” genes). and that of the POL group is 19±6 min [26] (the “lag” genes). The presence of lead-lag relationships between transient sub-complexes is briefly discussed in the supporting information file Text S1.

### Conclusions

The expression of genes in the cell is to a large extent controlled at the level of mRNA accumulation. One key point in the analysis of gene expression dynamics is that mRNA abundance is determined by two regulated processes: transcription and degradation both specifically affecting transcript levels. Computational analysis of genome-wide expression time series has shown that clusters of co-expressed (*i.e.* simultaneously correlated) profiles often provide clues for the presence of common transcription factors regulating both genes. Such computational analysis (known as “clustering”) is very useful since it allows the prediction of the underlying regulatory actions based exclusively on the available gene expression data obtained from a given experiment. The rationale behind such belief is a sort of a “guilty by association” approach: genes' products appearing and disappearing at the same time are likely to have some common transcriptional regulation. Nevertheless, it may well be the case that the same transcriptional signal regulating two (or more) genes may yield quite different outcomes on each transcript. In fact, a number of biological events following transcription may selectively affect cytoplasmic mRNA abundance, such as, for example, the activity of the enzymatic machinery involved in mRNA processing and degradation. In order to address this issue, we provided a novel computational methodology that, based exclusively on the available gene expression data, is able to effectively predict co-regulation even with variation in the dynamic response due to mRNA stability differences. Moreover, our approach also captures the relation of simultaneous or time shifted co-expression so that it provides a single integrative general index – the *lead-lag R^2^*−able to uncover the presence of a common regulatory signal underlying gene expression time dynamics also at the post-transcriptional level.

In order to test the validity of our approach on real data, we used yeast genome-wide cell-cycle expression time series obtained by several independent groups using different synchronization methods. In fact, by doing so, we could integrate the available cell cycle data and obtain a much more reliable aggregated dataset. We considered those gene pairs with the highest lead-lag R^2^ values and found the prediction for the presence of a common transcription factor to be highly consistent with protein-DNA binding data (ChIP experiments). Our results clearly indicate that co-regulation is not generally equivalent to simultaneous expression.

We believe that the same analysis can be successfully used to predict post-transcriptional regulation, *i.e.* the presence of a common mechanism able to stabilize or de-stabilize specific transcripts, as for the members of the PUF proteins family [2]. Moreover, we envisage the possibility that our methodology could be used on different data and organisms and thus providing a computational support to the understanding of transcriptional and post-transcriptional networks, given the recent growing interest in the post-transcriptional regulation layer [1] of gene expression (miRNA) and its role in many diseases, such as cancer. Finally, the characterization of the replication complex in terms of lead-lag relationships among gene expression time profiles of its sub-complexes suggests the possibility that our analysis could be effectively used as a tool for predicting the formation of transient multiple protein complexes.

## Materials and Methods

### Computation of the Simultaneous and Lead-Lag R^2^ Between Gene Expression Time Profiles

The mRNA relative abundance time course data obtained from cell populations experiments for gene A and B is denoted by *m_A_* and *m_B_*, respectively. The *simultaneous R* ^2^, is the usual squared Pearson correlation coefficient which measures the fraction of the total variance explained by a linear fit between the two variables *m_A_* and *m_B_*, that is
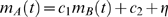
where *η* accounts for intrinsic and extrinsic noise.

The rationale behind the *lead-lag R* ^2^ is the following. We considered two genes, *A* and *B*, subject to the same regulatory signal (promoter activity) – possibly of different strength – due to the presence at their promoters of the same TF complex in its active state. Moreoever, we assumed that the change in mRNA levels due to the degradation rate could be reasonably well captured by a first order rate kinetics [53], and consequently the dynamic equation that includes both synthesis and degradation is the following
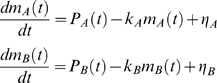
(1)where the two variables *m_A_* and *m_B_* measure gene expression on a linear scale (fold induction), *P_X_* is the promoter activity time profile of the TF complex relative to gene *X*, *α_X_* is its maximal strength, *k_X_* is the degradation rate (*k_X_* = log(2)/*t_1/2_*) and *η_X_* accounts for intrinsic and extrinsic noise. In order to remove size effects, the common signal between the promoter activities of the two genes is indicated as *p*(*t*) and is such that
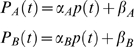
so that we get
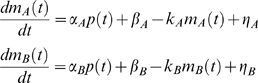
(2)From the second equation of (2) we have
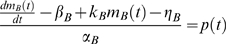
and substituting it into the first equation of (2) we obtain
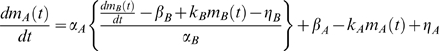
that can be rewritten as
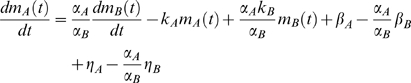
By evaluating the time integral of both sides we finally get:
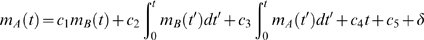
(3)where
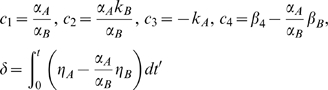
and coefficient *c_5_* accounts for the integration constant. The lead-lag *R* ^2^ is the fraction of the total variance explained by model (3). Note that the lead-lag *R* ^2^ depends on the time order of the data, whereas the simultaneous *R* ^2^ remains the same after a time shuffling of the data. Moreover, it is worth emphasizing that model (3) may well describe other biologically relevant mechanisms, such as time-shifted profiles as shown in the supporting information file Text S1. In this case, obviously, the coefficients *c_i_*, which depend on the underlying model, will change accordingly. Any pair of time profiles, satisfying model (3) will be said to have a lead-lag relationship and a good fit to (3) can be obtained also in situations different from those assumed to derive it. This property is very useful since it provides flexibility in modeling different biological phenomena resulting from the presence of a common regulatory signal.

The reason for the term “lead-lag” is due to the fact that two signals satisfying model (3) also define the transfer function of a “lead-lag compensator” widely used in control systems engineering. Assuming, for the sake of simplicity, the signals devoid of linear trends and noise (*c*_4_ = *c*_5_ = *δ* = 0), model (3) in the Laplace domain is as follows:
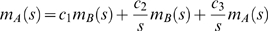
which can be rewritten as:
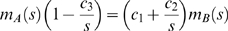
so that the resulting transfer function between *m_A_*(*s*) and *m_B_*(*s*) is that of a lead-lag compensator:
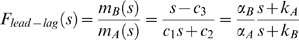



### A Direct Formula for Computation of the Lead-Lag R^2^ from Gene Expression Data

Let the available experimental time series of two genes *A* and *B* be composed of *N*>5 samples taken at times *t_1_*,…,*t_N_*. Model (3)
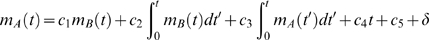
can be rewritten, using matrix notation, as follows
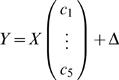
where Δ collects all of the noise terms and
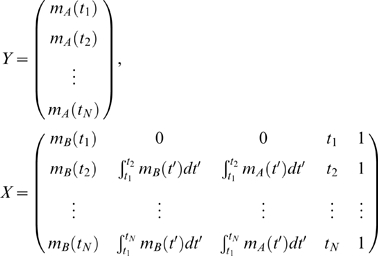
so that the least square estimation of the parameter vector is
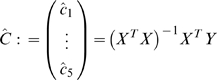
Accordingly, the goodness of fit to model (3) is measured by
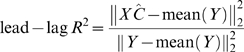
where the norm used is the usual Euclidean norm. It is important to note that the lead-lag R^2^ can be computed directly from gene expression data and values near unity indicates that the model well fits the available time series.

#### Numerical computation of time integral

Given a gene expression time profile [*mRNA*]*^t^* measured at times *t_1_*,…,*t_N_*, we computed its time integral in two steps. First, we used a piecewise cubic Hermite interpolation formula to obtain, for each time interval, 4 more samples. Over the interpolated time series we computed the integral by using a 2-points closed Newton-Cotes formula (trapezoidal rule).

### Datasets

#### Cell cycle regulated genes

We considered the extended list of 1159 cell cycle regulated genes reported in reference [38]. Each gene in this list has been considered as cell-cycle regulated in at least one of the six methods reported in reference [38]. We used such an extended list in order to have a sufficiently large dataset for our statistical analysis.

#### Gene expression datasets

We considered yeast cell cycle data measured by three independent groups [4],[43],[54]. The data from the Spellman *et al.* group consist of genome-wide gene expression data during the yeast cell cycle using three different synchronization methods. We denoted as ELU, the elutriation based dataset composed of one cell cycle, as ALPHA, the pheromone α arrest factor based dataset composed of two cell cycles and as CDC15 the temperature sensitive CDC15 mutant based dataset composed of three cell cycles. Only two cell cycles of the CDC15 dataset could be used due to the large number of missing data. The dataset in Cho *et al.* [54], denoted by CDC28, is composed of two cell cycle and synchronized using a temperature sensistive CDC28 mutant. The last dataset has been downloaded from the authors website [43] and is composed of three genome-wide gene expression measurement during the yeast cell cycle using alpha factor synchronization. We denoted such dataset, composed of two cell cycles each, as ALPHA_28, ALPHA_30 and ALPHA_38. Two data sets, ALPHA_30 and ALPHA_38, are dye swap technical replicates.

#### Transcription factors dataset

We considered the main cell cycle TFs (SWI4, SWI6, MBP1, NDD1, FKH1, FKH2, MCM1, ACE2, SWI5, YOX1) according to Bahler [39], and as targets, those genes included in the McIsaac *et al.* dataset [15] with a stringent threshold for DNA binding (*p*-value<0.001). The MacIsaac *et al.* dataset contained 660 of the 1159 cell cycle regulated genes. Therefore, we ended up with a list of 660 genes available for the subsequent computational analysis.

#### Half-lives dataset

We used half-life genome-wide measurements of the yeast transcript measured by Wang *et al.* [26] and by Kuai *et al.* [45].

#### Integration of gene expression datasets

For each dataset, we computed the simultaneous and lead-lag–*R* ^2^ for all possible pairs using *N* = 660 genes, that is we computed such parameters for *N*(*N*−1)/2 = 217470 pairs. More precisely, the *R* ^2^ values were computed for each cell cycle in each dataset, thus obtaining 13 values for each gene pair (ELU: 1 cell cycle, ALPHA: 2 cell cycles, CDC15: 2 cell cycles, CDC28: 2 cell cycles, ALPHA_28: 2 cell cycles, ALPHA_30: 2 cell cycles and ALPHA_38: 2 cell cycles). The average dataset has been constructed by computing the R^2^ values for each cycle and for each dataset, for a total amount of 13 cycles. The mean R^2^ value for each genes pair was obtained by computing the mean of the 13 available values. In case of missing data in the original dataset, computation of the mean R^2^ value was performed only when at least 8 out of 13 cycles were available. Such data were used to compute the diagram showed in Figure 3B. The values obtained by averaging all 13 cycles provided us with a single value for each gene pair and they were used to compute the ROC curve shown in Figure 3A. Cell cycle data with missing values were removed from the dataset.

## Supporting Information

Text S1 Supporting Information file
(0.10 MB DOC)
