# Supplementary material for: Embedding mRNA Stability in Correlation Analysis of Time-Series Gene Expression Data
Source: PLoS Comput Biol. 2008 Aug 1;4(8):e1000141. doi: 10.1371/journal.pcbi.1000141 (PMC2453326; doi:10.1371/journal.pcbi.1000141)
Supplement: Text S1 — Supporting Information file (0.10 MB DOC) [file pcbi.1000141.s001.doc]

**Supporting information**

*Embedding mRNA stability in correlation*

*analysis of time-series gene expression data*

*Lorenzo Farina, Alberto De Santis, Samanta Salvucci, Giorgio Morelli and Ida Ruberti*

*ROC curves using different p-values for binding and conservation criteria*.

Here we show the ROC curves computed as in Figure 3A using all the available conservation criteria considered in MacIsaac  *et al*, 2006.

Conservation criterium: *p*-value for binding 0.05; no phylogenetic conservation:

Conservation criterium: *p*-value for binding 0.05; 1 level of phylogenetic conservation:

Conservation criterium: *p*-value for binding 0.05; 2 levels of phylogenetic conservation:

Conservation criterium: *p*-value for binding 0.001; no phylogenetic conservation:

Conservation criterium: *p*-value for binding 0.001; 1 level of phylogenetic conservation:

Conservation criterium: *p*-value for binding 0.001; 2 levels of phylogenetic conservation:

*Equations used for the simulations shown in Figure 1*

The abundance of mRNA is determined by two regulated processes: transcription and degradation, both specifically affecting transcript levels. Formally, we can describe the rate of change of each mRNA species *m*(*t*) as the result of a time-varying rate of transcription function *T*(*t*) and a time-varying rate of degradation function *D*(*t*) as follows

$$\frac{dm(t)}{dt}=T(t)-D(t)$$

The simulations shown in Figure 1 has been performed by assuming a first order kinetics for the transcript degradation process

$$\frac{dm(t)}{dt}=T(t)-k(t)m(t)$$

where $T(t)$ is the transcription rate and $k(t)$ is the degradation rate.

*Lead-lag relationship and dynamic protein complex formation*

Here we show, using a simple mathematical model, that mRNA time profiles of genes encoding two subunits of a protein complex are in principle related by a lead-lag relationship. To see this, we follow the reasoning of Jansen *et al.*, 2006, and consider a kinetic model of protein and mRNA concentrations during the formation of a protein complex. Let $p_{i}(t)$ and $p_{j}(t)$ be the protein concentrations of two different subunits of a protein complex, and $m_{i}(t)$ and $m_{j}(t)$ the mRNA concentration of the corresponding gene. Then, we consider a first order model as follows

$$\begin{matrix} \frac{dp_{i}(t)}{dt}=k_{m_{i}}m_{i}(t)+k_{p_{i}}p_{i}(t) \\ \frac{dp_{j}(t)}{dt}=k_{m_{j}}m_{j}(t)+k_{p_{j}}p_{j}(t) \end{matrix}$$

where $k_{m}$ is an mRNA traslation rate constant and $k_{p}$ is a protein degradation constant. The two equations can be rewritten in the Laplace domain as

$$\begin{matrix} sP_{i}(s)=k_{m_{i}}M_{i}(s)+k_{p_{i}}P_{i}(s) \\ sP_{j}(s)=k_{m_{j}}M_{j}(s)+k_{p_{j}}P_{j}(s) \end{matrix}$$

where $P(s)$ and $M(s)$ are the Laplace transforms of the protein and mRNA concentration time profiles. By straightforward algebraic manipulations we obtain

$$\begin{matrix} M_{i}(s)=\frac{s+k_{p_{i}}}{k_{m_{i}}}P_{i}(s) \\ M_{j}(s)=\frac{s+k_{p_{j}}}{k_{m_{j}}}P_{j}(s) \end{matrix}$$

so that we can write the relationship, in the Laplace domain, between the time profiles of the mRNA concentrations of the two subunits of the protein complex

$$\frac{M_{i}(s)}{M_{j}(s)}=\frac{k_{m_{j}}}{k_{m_{i}}}\frac{s+k_{p_{i}}}{s+k_{p_{j}}}\frac{P_{i}(s)}{P_{j}(s)}$$

Therefore, the above formula establishes a lead-lag relationship between $m_{i}(t)$ and $m_{j}(t)$and this is a formal explanation of the high lead-lag R^2^ values observed for gene expression time profiles of the genes coding for subunits of the replication complex shown in Figure 7.

*List of best pairs exceeding the 95^th^ percentile*

The list of gene pairs whose lead-lag R^2^ value is larger than the 95^th^ percentile of the overall distribution can be found, in excel format, at the address:

<http://www.dis.uniroma1.it/~farina/leadlag/leadlag.xls>

*Time-delayed profiles and lead-lag R^2^*

Here we show that the lead-lag R^2^ is able to capture the presence of a time-delayed profile in an approximate but efficient way. In fact, in general, the problem of finding the optimal time-delay between two given profiles is combinatorial in nature since one has to search among all possible delays or using local clustering techniques (see Qian *et al.*, 2001) which are able to reduce to some extent the inherent problem complexity. To see how the lead-lag R^2^ may approximately describe shifted time profiles we first assume that the mRNA signal is sinusoidal. In this case we have:

where *T*  is the time period of the sinusoid and $t_{s}$ is the time-delay. Using a well known trigonometric formula we get

$$\sin\left(\frac{2\pi}{T}\left(t-t_{s}\right)\right)=a\sin\left(\frac{2\pi}{T}t\right)-b\cos\left(\frac{2\pi}{T}t\right)$$

where$a=\cos\left(\frac{2\pi}{T}t_{s}\right)$ and $b=\sin\left(\frac{2\pi}{T}t_{s}\right)$. Therefore we have

$$m_{A}\left(t\right)=c_{1}m_{B}\left(t\right)+c_{2}\int_{ 0}^{ t} m_{B}\left(t'\right)dt'$$

since

$$\cos\left(\frac{2\pi}{T}t\right)=-\frac{T}{2\pi}\int_{ 0}^{ t} \sin\left(\frac{2\pi}{T}t'\right)dt'=-\frac{T}{2\pi}\int_{ 0}^{ t} m_{B}\left(t'\right)dt'$$

Consequently, the lead-lag R^2^ of two time-delayed sinusoids is equal to its maximal value, *i.e.* to 1. In this case, the presence of a delay is perfectly detected without error. By contrast, in the case of a generic profile (*i.e.* not well described by a sinusoid) we show hereafter that still a time-delayed profile can be described by our model. In fact, in the Laplace domain, a time-delayed relationship is the following

$$m_{A}(s)=e^{-Ts}m_{B}\left(s\right)$$

Since the lead-lag relationship in the Laplace domain is a rational function, we consider the widely used first order Padé approximant. The Padé approximation of the delay in the Laplace domain is given by

$$m_{A}(s)=\frac{1+as}{1+bs}m_{B}\left(s\right)$$

where

$$a=-\frac{T}{2}, b=\frac{T}{2}$$

so that we end up with a lead-lag relationship which is an optimal first order Padè approximation of a time-delayed relationship. Note that, such approximation procedure can be applied also in the presence of a time-warp between the delayed time profiles by simple rescaling of the coefficients.
